# Hospital and Institutional News

**Published:** 1915-05-29

**Authors:** 


### HOSPITAL AND INSTITUTIONAL NEWS.

### THE NEW KING GEORGE HOSPITAL OPENED.

On Wednesday, the earliest date on which any wards could be made available, the fourth and fifth floors of the King George Hospital in Stamford Street, Waterloo Road, were opened for the reception of sick and wounded soldiers. By this means some 630 beds have become immediately available, in response to an urgent request from the War Office. In accordance with military custom there was no formal opening, but the building will come into use by instalments as each section of the institution (which, it will be recalled, was designed for the new Stationery Office) becomes equipped. This work of equipment, undertaken by the joint societies, is being pushed forward as fast as possible throughout the whole building. As soon as the structural alterations required by the War Office are complete the three remaining ward floors, the ground floor, the basement, and special departments will be furnished and occupied in a similar manner. When this is done the vast hospital will contain 1,650 patients. It may be added that a Compassionate Fund has been started, with the approval of the Director-General, to provide additional comforts and clothing, and also to equip men discharged from the Service as medically unfit with such clothes, tools, or surgical instruments as may aid them either to resume their former occupations or to make a new start. The fund will supplement, but, of course, not supersede, Government allowances and existing forms of relief. Another branch of the Compassionate Fund is the gift stores. All gifts to patients, in accordance with the rules officially approved, will be pooled, and each ward sister will make requisitions for individual patients. The wishes of individual donors, however, will be carefully considered, but the aim throughout is impartial distribution.

### HOSPITALS AND THE SPIRIT TAX.

The difficulty with regard to the surtax on medicinal spirits has now been solved by the inclusion in the Finance Bill of a sub-clause which provides for the repayment of the surtax on spirits used in the manufacture of medicines. The provision is as follows: "If any person proves to the satisfaction of the Commissioners of Customs and Excise that any spirits to which the restrictions contained in the Immature Spirits (Restriction) Act, 1915, do not apply, have been delivered to him and used solely in the manufacture or preparation of any article recognised by the Commissioners of Customs and Excise as an article used for medical purposes, or have been used for scientific purposes, that person shall be entitled to obtain from the Commissioners repayment of the amount of duty (if any) paid under this section in respect of the spirit used." Hospitals will therefore be required to pay a surtax on rectified spirit, but on proof that it has been used for medical purposes the amount of the surtax will be refunded. The freeing of medicinal spirits from this extra tax appears to have been largely due to the efforts of Mr. W. S. Glyn-Jones, M.P., the Pharmaceutical Society's Parliamentary Secretary, who placed the case for chemists and doctors and public institutions before the Government very forcibly.

### THE FULHAM MILITARY HOSPITAL.

The Fulham Military Hospital—the second of the London Poor-Law infirmaries to be handed over to the War Office for the use of sick and wounded soldiers—is now ready for its new work, and is receiving batches of wounded daily. The medical superintendent, Dr. C. Thackray Parsons, has been appointed officer in charge, with the rank of major. The medical staff consists of five lieutenants of the R.A.M.C., six half-time medical men, a consultant staff of two, a radiographer, and a pathologist. Amongst the staff are Mr. H. Chapple, surgeon to Guy's Hospital; Mr. Howell Evans, assistant surgeon to the Cancer Hospital; Lieutenant Ralph Williams, Senior Assistant Medical Officer, Board of Education; Dr. S. Henning Belfrage, Dr. H. Chapple, Dr. J. Gay, Dr. A. C. Gray, Mr. H. G. Wharry, and Lieutenants H. Cumming, R. Dainty, John Forbes, and W. J. Hill. Miss Florence Stoney is the radiographer, Captain Davis the pathologist, Mr. Lockhart Mummery consulting surgeon for diseases and injuries of the intestines, and Dr. S. A. Kinnier Wilson consulting neurologist.

### POOR-LAW MEDICAL OFFICERS AND CERTIFICATES UNDER THE MENTAL DEFICIENCY ACT.

Owing to the refusal of the Leeds Mental Deficiency Committee to pay a fee to the medical officer of the Hunslet Workhouse for a certificate under the Mental Deficiency Act given at their request, the Guardians asked the Local Government Board to decide by whom the fee should be paid. The matter was referred to the Board of Control, which expressed the opinion that the certification by a medical practitioner is one of the steps essential for dealing with the case of a defective who is to be sent to an institution or placed under guardianship in accordance with the Mental Deficiency Act, and that the cost of the certificate should be borne by the local authority. In forwarding this letter to the Guardians, the Local Government Board stated that they have no jurisdiction at the present stage to determine the proper incidence of the charge, but they see no reason, as at present advised, to differ from the view of the Board of Control. It is satisfactory to find that certification under the Mental Deficiency Act is not one of the duties resulting from recent legislation which can be demanded gratuitously from the Poor-Law medical officers.

### THE CRUCIFORM BLOCK.

The new buildings at the Cambridge Borough Isolation Hospital have now been opened. When the borough was extended in 1912 the accommodation which had up to that time been provided had grown from the still remaining rudimentary buildings of 1884. It was pointed out at the opening that when the new administrative block had been opened in 1899 no addition was contemplated. But since then, the Chairman of the Public Health Committee remarked, the prevalence of diphtheria had increased, and there had been a change of type in scarlatina, which had become mild indeed, but also increasingly prevalent. The buildings had therefore been overcrowded, and when the borough extension was arranged for further accommodation became imperative. The administrative block has been extended, with more adequate provision for the staff, and the two new blocks provide twenty-six beds. One of the blocks is cruciform and contains twelve beds separated by glass partitions, with a duty room in the centre for the nurses. The architect is Mr. E. Jenkins Chart, of Croydon, who has specialised in this design of isolation hospitals. It was pointed out that this system had proved successful in the Croydon rural district, but that its disadvantage was in respect of the ventilation of individual wards, and on this account the design of the cruciform block had been evolved. Sir Clifford Allbutt has described the buildings as admirable, and pointed out that they also remove the local complaint of insufficient accommodation.

### DEATHS FROM STARVATION.

Mr. J. Theodore Dodd, in a letter to the Press, draws attention to the striking variation in the incidence of the deaths from starvation in the various parishes and unions of the County of London. He points out that the starvation death-rate per million for the three years 1904-06 was, for London as a whole, 30; for the Eastern District of London, 79; Whitechapel, 291; Poplar, 29; Stepney, 86; St. George-in-the-East, 40; and Mile End, 8. For the seven years 1907-13 the rate was: Whitechapel, 700; Poplar, 0; Mile End, 31. The large excess in Whitechapel calls for a searching investigation. There poverty will not explain it, for the conditions in Whitechapel are no worse than in Poplar and Mile End. The explanation is probably to be found in the system under which Poor-Law relief is given in Whitechapel. A system which produces such results stands condemned, and it is the duty of the Guardians to examine the rules under which relief is given and to render them less harsh and deterrent.

### DISPENSING IN MENTAL HOSPITALS.

Following on the recent death by accidental poisoning at the Royal Bethlem Hospital and the recommendation made by the Coroner at the inquest, the appointment of Mr. J. Oliver Thomas, chemist and druggist, as dispenser will be interesting to hospital officers. Mr. Thomas holds the post of pharmacist to the Royal South London Dispensary, and it is understood that he will now hold both appointments. Gradually the dispensing arrangements of mental hospitals are being improved, but there is still a significant exception to this advance in respect of the State criminal asylums at Broadmoor and Rampton. According to the Civil Service estimates it appears that neither of these institutions carries a pharmacist on its strength. At Rampton there is shown an item of £10 per annum, paid additionally to an attendant for dispensing; whilst at Broadmoor the accounts are silent altogether about the subject, the presumption being that the work of dispensing is done by one of the medical officers. Such arrangements are fair neither to the institution, the staff, nor the patients; and when the State itself is the culprit, then the fault is even greater.

### VOLUNTARY AID IN MENTAL DEFICIENCY WORK.

After correspondence with the Board of Control, the London Mental Deficiency Committee is proposing that a grant shall be made to the London Association for the Care of the Mentally Defective on the understanding that that body undertakes a part of the work which falls upon the London County Council under the Mental Deficiency Act. The only work which it seems possible for the Council to delegate to the Association is the duty of ascertainment of defectives; of supervising defectives who are subject to be dealt with under Section 2 (1) (*b*) of the Act and are not in institutions or under guardianship; and that of finding guardians for suitable cases. As regards ascertainment, the committee are of opinion that this important duty, upon which so much will depend, should not be delegated to a voluntary association but should be left to the officers of the local authority, with their special knowledge and experience. It is therefore intended to delegate to the Association the two duties of supervision and of finding suitable guardians, and the amount of the grant suggested is £200 a year. The Board of Control is also to be asked to make as generous a grant as they reasonably can to the work of the Association, having regard to the difficulty which is likely to be experienced just now in procuring voluntary subscriptions.

### LOCAL AUTHORITIES AND VOLUNTARY AID.

During the correspondence with the Council on this subject the Board of Control have laid down certain axioms which should be of great value to other bodies undertaking this work. The Board say that while they recognise that the Act gives no explicit directions as regards the power of a local authority to contribute towards the expenses of a voluntary society incurred in assisting or supervising defectives for whom the local authority is not responsible, the intention of the Act is not to debar a local authority from supervising them. While, therefore, the Board could not authoritatively determine the question of law they would hold that a local authority in certain circumstances might properly supervise defectives or alternatively authorise a voluntary society to do so and contribute towards the expense. A voluntary society, of course, might carry out this work without express delegation from the local authority, but experience has shown the Board that societies are anxious to obtain the recognition and approval of both the central and the local control authority, and that the work of societies is greatly facilitated by such guidance and control.

### THE SECRETARYSHIP OF HAMPSTEAD GENERAL HOSPITAL.

We are asked to say that, owing to the enormous number of replies which have been received to the advertisement for the vacant post of secretary, it has been found quite impossible to acknowledge them individually. The applications are at present being sifted by the selection committee, and selected candidates will be duly informed of the date on which they are desired to attend at the hospital. Others are desired to note that there is no discourtesy intended in not acknowledging their applications, as the clerical work alone involved is beyond the scope of the present clerical staff of the hospital.

### A "SPORTING SPIRIT" IN RELIGION.

It is not often that matters of religion, or rather denominational concerns, are brought before an annual meeting, but a remarkable practice was mentioned at the Preston Royal Infirmary meeting last week by the institution's chaplain, Rev. A. C. Schofield. He referred to the visit of infirmary secretaries to the institution some time ago, and remarked that one of the few criticisms made on that occasion was that labels above the beds defining the religion of the patient should be removed. Mr. Schofield thought it a wise practice; "it prevented any excuse." They required in religion, he added, a real sporting spirit, as in other matters, and it did not seem to him a sporting action to try to rouse a man, while lying on his back, to change his religion. It was tried on one man, but there was no excuse for it. To claim patients for this or that denomination cannot be the proper work of any hospital chaplain, and surely there must be something wrong with the chaplains of any institution who require to be "warned off" in the manner suggested. Visiting is part of a chaplain's duty, and he may easily be of much service to patients whose denomination is not that which he represents. But while the great opportunity of visiting the sick is a virtue or recognised work of mercy which any devout chaplain will regard as a Christian privilege to perform, the thrusting on those he visits of dogmatic questions or sacramental offices without first ascertaining that his patient desires them is a breach not merely of his duty, but of Christian charity as well. We therefore must conclude that the use of labels defining the denomination of each patient is undesirable, since any chaplain who requires their aid to prevent him from "claiming" the patient is quite unfitted to perform this valuable and delicate work.

### THE TROOP TRAIN DISASTER.

The three trains which collided last Saturday on the Caledonian Railway at Quintin's Hill, a mile north of Gretna Green, have apparently been involved in the worst disaster in the history of British railways. An official statement issued on Monday gave the number of killed as 157 and the number of injured as 200. To add to the horror of the circumstances many were burned alive, and the heaviest toll of victims fell upon the crowded occupants of the troop train. Mr. Donald Mathieson, general manager of the Caledonian Railway Company, has summed up the bare facts of the accident by stating that a slow train from Carlisle was shunted from the down to the up line, where, while stationary, it was run into by the south-going troop train, which was carrying 500 officers and men, and immediately following this collision the express train from Euston dashed into the wreckage. The troop train almost immediately caught fire, and the flames prevented the rescuers from removing many of the soldiers, who were either helpless from injury or pinned beneath the shattered carriages. Fire engines arrived from Carlisle, and the flames, save where the engines were burning, were eventually quelled. Medical aid was telephoned for and arrived from Gretna and Carlisle, first-aid being given in a field hard by the scene of the disaster. The Cumberland Infirmary, Carlisle, was unequal to all the demands upon it, but it rose heroically to the occasion and admitted 74 cases. It possesses 154 beds, of which 102 are available. The Board of Trade inquiry was opened at Carlisle on Tuesday by Lieutenant-Colonel E. Druitt, R.E., Inspecting Officer of Railways.

### A FARTHING RISE IN THE COST OF PROVISIONS.

It is not often that questions are asked in committee on the ground that the cost of provisions is too low; but at the quarterly meeting of the Walsall Hospital the question was raised as to how the cost of provisions had risen only one farthing per head. It was pointed out that the number of the staff was exactly the same as for the corresponding period for 1914; and that, while the figure would be a matter for congratulation at ordinary times, it was infinitesimal considering the extraordinary increase in the cost of commodities. It seemed, said the speaker who raised the point, highly out of proportion, even if the provisions were contracted for. The Chairman then explained that the hospital's provisions were purchased by contract, and that the contracts were made last June and terminated this June. Thus, with very few exceptions, the prices of goods are those which prevailed twelve months ago. Testimony was then paid to the quality of the goods; but this really did not arise in view of the Chairman's explanation. It is satisfactory to note that the contractors in this instance have proved not only loyal to their bargains, but apparently uncomplaining; for, where they have been hard hit by the war, hospital administrators, once the position was explained and the willingness to co-operate manifested, have been prepared to meet them. A mutual arrangement has then been made, and we hope that the new contracts to be made at Walsall will be drafted in this spirit, and thus a singularly happy passage through the crisis be continued in what will soon be the second year of the war.

### THE WORK OF THE SPECIAL HOSPITALS FOR OFFICERS.

An interesting statement of progress has been published in connection with the treatment of traumatic neurosis or battle shock, which led to the opening of the special hospital for officers at 10 Palace Green, Kensington. Suggested by Dr. Maurice Wright, the sum of £10,000 was subscribed and handed over to Lord Knutsford, who now reports what has already been done. Since the special hospital became known, it has, he says, always been full, and more than one hundred officers have passed through it who, with few exceptions, have recovered. Acting for the Director-General, Dr. Aldren Turner has completed a series of arrangements by which officers are sent straight from the Front to the special hospital. This organisation is mentioned as having been very helpful in restoring the officers to health. How much the work is appreciated is to be inferred from Lord Knutsford's statement that a second special hospital has been opened. It will be remembered that complete quiet and isolation are essential to such institutions, and to find sites and buildings with these characteristics is not a very easy matter in London. However, Mr. R. L. Harmsworth generously gave his furnished house, Moray Lodge, Campden Hill —a large house standing in four acres or more of grounds. This, we learn, has now been opened for thirty-three patients. It is understood that there is enough money in hand to run the two hospitals for nine months more.

### INCREASING THE STAFF AT HOPE HOSPITAL.

Some important recommendations with reference to the staff of the Hope Hospital have been adopted by the Salford Board of Guardians on the recommendation of the infirmary visiting committee. The increases to the staff sufficiently point to the amount of work which is being undertaken. Thus the matron recommended the permanent appointment of a second assistant matron and of a second night superintendent. As to the staffing of the infirmary during its occupation by the sick and wounded soldiers, the committee has decided to engage eleven trained and certificated nurses at a salary of £36 per annum. It was left to the executive committee to engage additional help from time to time, since, if the number of beds actually set aside for wounded soldiers were filled, the appointment of a number of untrained nurses would be necessary. After some discussion it has been decided that the appointments should be made only for the duration of the war. In regard to the expense, the War Office has notified the Clerk that if the Guardians spend more than the definite sum allowed per head for the cost of wounded soldiers, the claim for an additional grant would be favourably considered.

### WOMEN DOCTORS AFTER THE WAR.

We have always preferred to believe that the door which the European War has opened to medical women will not be closed to them once peace is declared, though it may be still necessary to warn extreme enthusiasts that matters may not prove quite as simple as they suppose. We, therefore, welcome the address given last week by Miss Brooks, Secretary and Warden of the London (Royal Free Hospital) School of Medicine for Women, on the opportunities of women doctors. She said that it was an error to suppose that medical women were the product of the war, which had served merely to single out its most brilliant members. In private practices, especially in the country, where their work was much appreciated, there was, she said, a great demand for women doctors. As to their position in London, she pointed out that women were at work in half-a-dozen London hospitals, and it was to be hoped that the positions of visiting physician and surgeon would soon cease to be restricted to men, and would be opened to women, especially in hospitals for women and children. On the broader question of the future, Miss Brooks remarked that women were badly needed to attend infants and young children suffering from the diseases peculiar to them. She then went on to point out the need for the establishment of infant clinics throughout the country. The argument underlying these remarks, which may, perhaps, be summarised as the need for better medical treatment of various types of disease which are not reached by our existing medical organisation, and the fitness of medical women to undertake the new extension of work, seems to us far-sighted and practical. This point of view depends, not upon a desire to exclude medical women from existing branches of professional work, but on the fact that one of their principal virtues lies in an aptitude for a necessary type of medical work which so far has not been adequately undertaken. The demand for them created, in a new sense, by the war, will be amplified in new fields after it is over.

### HUT-WARDS IN LONDON.

The pressure on our hospital accommodation, and the efforts made by the voluntary hospitals to relieve it without diminishing their beds for civilians, could hardly be better illustrated than by the huts which are being built at St. Thomas's Hospital. These huts, which are nearly ready for opening, will accommodate 300 additional beds for sick and wounded soldiers. In order to make them comfortable for their patients the public is being asked by the staff to furnish them with such luxuries as easy-chairs and cushions, books and flowers. Some information has already been published in our columns concerning different types of emergency or temporary wards in the provinces, and, together with the open-air hospitals which have also been adopted here and there, it is evident that the war will leave its mark upon hospital design and architecture. In view of the prevalence and necessity of adapted or improvised buildings, in which good work is undoubtedly being done, the tendency toward architectural magnificence, especially in those hospitals with medical schools, may receive less support in future years. The adoption of the new huts at St. Thomas's Hospital is therefore suggestive in more ways than one, and the young hospital architect of the present day should keep his eyes open for all the lessons that are being learnt from these and many similar constructions.

### A NEW MEDICAL POST.

For some time past the London Insurance Committee has had under consideration the question of appointing a full-time medical adviser. Hitherto Dr. J. Edward Squire has acted as part-time medical adviser to the Committee, but of late the work has increased so rapidly that it was felt that some alteration in the system was necessary. The Committee have now prepared a list of the duties which such a whole-time adviser would be required to carry out. They include the classification of cases to be recommended for medical benefit, the visiting of tuberculosis dispensaries and any other institutions with which the Committee may have agreements, and a variety of other duties. It has accordingly been decided to appoint a full-time adviser, and the post has been offered to Dr. Squire at a salary of one hundred guineas a month. Dr. Squire will be debarred from doing general work, but it is suggested that he shall be allowed to retain a position which he at present holds as consulting physician to a life assurance society.

### THE NUMBER OF DOCTORS ON WAR SERVICE.

After all the consideration given to the shortage of doctors it is interesting to have the figures of those engaged on war service, as given in the House of Commons by Mr. Tennant in reply to a question from Mr. Joynson-Hicks. These figures state that there are at present 7,027 medical men serving, including 1,008 in the Army Medical Service and the Royal Army Medical Corps. The re-employed retired R.A.M.C. officers number 174; there are 623 Special Reserve R.A.M.C. officers, while the number of temporarily commissioned officers is 3,100. The Territorial Force R.A.M.C. numbers 2,122. It is interesting to learn that over 5,000 medical men have offered their services, of whom 3,100 have been actually appointed. Mr. Tennant also stated that there was at the moment no scarcity of medical officers, either at home or at the Front, but that a further large number would be required. This somewhat ambiguous statement does not throw much light upon the actual condition of affairs, but since military reasons do not preclude numbers being given, we must hope that when " a further large number " is required the precise number will be asked for. It has been the lack of precision in the demands for more doctors that has made it so difficult to mobilise the profession to meet the military and civil needs of the war.

### GUARDIANS AND THEIR SUBSCRIPTIONS TO HOSPITALS.

The vexed question of the subscriptions to hospitals from Metropolitan Boards of Guardians has been referred to in these columns from time to time, and an analysis of the donations of the Lambeth Board of Guardians for the year, which have recently been adopted, is worth consideration. The Lambeth Guardians have resolved to distribute £149 13s. amongst a variety of organisations which assist them in their work. These include five hospitals, which receive between them £36 10s., the recipients being Brompton Hospital, £10 10s.; Cancer Hospital, £5; Royal Eye Hospital, £5 5s.; Royal Mineral Water Hospital, Bath, £12 12s.; and St. John's Hospital for Diseases of the Skin, £3 3s. Nursing associations receive amongst them £26. The balance of the subscriptions amounts to £77 3s., and this is divided between organisations befriending boys, girls, and servants, such as the Church Army, the National Society for the Prevention of Cruelty to Children, and societies dealing with the blind. It is doubtful if these annual subscriptions of £36 10s. represent a fair percentage of what is due to the hospitals, and even if £26 for nursing is a sufficient amount, when it is remembered that by nursing the sick poor in their own homes the societies are keeping the people out of the infirmaries. A subscription from a public authority is not really a satisfactory or businesslike way of meeting its obligations. What undisputed and direct assistance the Guardians receive should be paid for by arrangement between the organisation on which they rely and the board.

### EXTRAORDINARY EXPENDITURE IN WAR-TIME.

Much of the art of hospital finance consists in bringing ordinary and extraordinary expenditure into intimate relation with each other by averaging the latter and spreading its burden equally over different years. Some of the bigger hospitals even average their appeals, but in the case of smaller institutions such methods of management cannot be so strenuously applied. During the war, then, the question of extraordinary expenditure becomes an acute problem. We note with interest that the Hon. Henry Hannen, recently appointed Chairman of the committee of management of the West Kent General Hospital, Maidstone, faced with this problem, has issued an appeal. The need is for structural repairs, due to the weather-worn condition of the pointing of the brickwork, with the risk of damp which it entails. The window-frames have also become dilapidated. Mr. Hannen's committee, therefore, have boldly appealed for £400. There ought to be no difficulty in raising this money. The war has revealed as many new sources of charity as it has exhausted others. Indeed, the real difficulty, as organisers of appeals know, is not so much to raise money from people who have proved their generosity as to induce new individuals to subscribe. There need, then, be no undue discouragement as to the risk of issuing an appeal in war-time. The war itself has been the best advertisement of the hospitals, and, commercially speaking, they stand to gain more from this publicity than to lose because much money has been given to other causes. Practically, moreover, the hospital serves Maidstone and some ninety parishes. That Maidstone cannot raise £200 for her hospital, and the parishes the remaining half, is incredible. We mention these facts to show how simple a problem a well-thought-out appeal may be, notwithstanding the war. This may encourage responsible hospital officers in other institutions also as to the way in which extraordinary expenditure should be faced.

### THE HOSTEL FOR BLINDED SOLDIERS.

In an official *communiqué* issued this week a well-deserved tribute is paid to the work of the Blinded Soldiers' Care Committee. It announced that the War Secretary has approved of the arrangements which have been made for providing additional accommodation at the Blinded Soldiers' Hostel at St. Dunstan's, Regent Park, to an extent which will enable 120 men to be cared for and trained there. These arrangements include the erection of spacious workshops, besides those already in use, and considerable additions of a temporary character to the house.

### BRITISH PHARMACEUTICAL CONFERENCE.

The 1915 meeting of the British Pharmaceutical Conference, which had been arranged to take place at Scarborough in July next, has been abandoned. The Conference Committee have now decided to hold a business meeting in London in July next. The joint honorary secretaries are Mr. Horace Finnemore, B.Sc., the chief pharmacist at Guy's, and Mr. R. R. Bennett, B.Sc., formerly chief pharmacist at University College Hospital, and now on the staff of a large firm of wholesale druggists. The Conference president this year is Captain Peck, M.A., Ph.C., of Cambridge.

### INCREASED DEMAND FOR PATENT MEDICINES.

Contrary to what was expected, the demand for patent medicines has increased since the Insurance Act came into force. This is clearly shown by the figures relating to the revenue from medicine stamp duty contained in the latest Report of the Commissioners of Customs and Excise. During the year ended March 31, 1914, the revenue was £360,377; whereas in 1910 the revenue was £313,114; in 1911, £325,646; and in 1912, £327,857. Compared with the year 1910 the increase is about £47,000 sterling. The duty on proprietary and secret remedies of the selling value of a shilling and under is three-halfpence, so that if, for the purpose of a rough estimate, it were assumed that all medicines were of that value, the increase in the revenue would represent an increase in sales of over seven million packages, of a total value of more than three-quarters of a million sterling. The fact thus emerges that although insured persons can obtain proper medical advice and attendance without extra payment, some of them still prefer to doctor themselves!

### SPECIAL HOSPITALS IN CANADA.

The recent severe losses to the Colonial regiments, and the extraordinary dash and courage displayed by them, have brought home to the English-speaking peoples overseas as nothing else could their part and lot in the great commonwealth we speak of as the Empire. Just as the railway disaster of last week, in which a Scotch regiment suffered so terribly, brought the war home to many whom events not on their own soil somehow fail to arouse, so the recent Colonial casualties, by the special institutional needs they have occasioned, have redoubled the patriotism of the Dominions. As an instance we may merely quote the decision recorded by the *Times* correspondent in Toronto to establish at Valcartier a hospital for members of the Canadian forces who have been incapacitated for further service. Other men are to be sent to convalescent homes in various parts of Canada, while men unable to earn a living will be pensioned at once. There is no doubt that the special institutions required for different classes of wounded men arouse as nothing else does a sense of the requirements which each Dominion has to meet, and confirm and strengthen the first wave of patriotism which led to enlistment. The institutional side of the problem which has now begun to come to the fore is, however, an abiding fact, and the new hospital at Valcartier will remain needed and at work after the war is over. It is inspiriting for hospital men to note the way in which the work of their institutions crystallises public feeling and strengthens the common purpose of the Empire to pursue the war to its end.

### THIS WEEK'S DRUG MARKET.

The intervention of Bank Holiday naturally disturbed the course of business, but a speedy recovery from the effects of the holiday may be expected. The fact that Italy is now at war will probably not have a very marked influence on the drug market; drugs of Italian origin are not numerous, and the action which Italy has now taken had been anticipated for so long that buyers had had time to make their arrangements accordingly; such articles as citric acid, essence of lemon, and oil of orange may probably advance in price, but the prohibition of the export of sulphur from Italy may not affect the value of this commodity very seriously, as there are other important sources of supply. Cream of tartar and tartaric acid have an advancing tendency in price. There is little change in the position of cod-liver oil. A good business continues to be done in quinine, and prices have an upward tendency. Epsom salts is very scarce, and the price is very much higher; in fact, it is very difficult to obtain quotations for large quantities. The reason of the scarcity is that the bulk of our supplies of magnesium sulphate had, until the outbreak of war, come from Germany, where the drug is manufactured from raw material obtained from the Stassfurt mines. Magnesium sulphate can, of course, be produced in this country, but the Government having commandeered the supplies of sulphuric acid, which is employed in the manufacture of Epsom salts, the position is a difficult one. It is not expected that freer supplies will be available for some time.

### TO OUR READERS.

Contributions are specially invited from any of our readers to these columns. They should deal with topical subjects and news. They must be authenticated for the information of the Editor only. The minimum payment if published is 5s. There is no hard-and-fast rule as to space, but notes of about twenty lines in length are preferred

